# Single Cell Transcriptome in Colorectal Cancer—Current Updates on Its Application in Metastasis, Chemoresistance and the Roles of Circulating Tumor Cells

**DOI:** 10.3389/fphar.2020.00135

**Published:** 2020-02-27

**Authors:** Francis Yew Fu Tieng, Rashidah Baharudin, Nadiah Abu, Ryia-Illani Mohd Yunos, Learn-Han Lee, Nurul-Syakima Ab Mutalib

**Affiliations:** ^1^UKM Medical Molecular Biology Institute (UMBI), Universiti Kebangsaan Malaysia, Kuala Lumpur, Malaysia; ^2^Novel Bacteria and Drug Discovery Research Group, Microbiome and Bioresource Research Strength, Jeffrey Cheah School of Medicine and Health Sciences, Monash University Malaysia, Subang Jaya, Malaysia

**Keywords:** single-cell RNA sequencing, colorectal cancer, metastasis, chemoresistance, tumor heterogeneity, circulating tumor cells

## Abstract

Colorectal cancer (CRC) is among the most common cancer worldwide, a challenge for research, and a model for studying the molecular mechanisms involved in its development. Previously, bulk transcriptomics analyses were utilized to classify CRC based on its distinct molecular and clinicopathological features for prognosis and diagnosis of patients. The introduction of single-cell transcriptomics completely turned the table by enabling the examination of the expression levels of individual cancer cell within a single tumor. In this review, we highlighted the importance of these single-cell transcriptomics analyses as well as suggesting circulating tumor cells (CTCs) as the main focus of single-cell RNA sequencing. Characterization of these cells might reveal the intratumoral heterogeneity present in CRC while providing critical insights into cancer metastasis. To summarize, we believed the analysis of gene expression patterns of CTC from CRC at single-cell resolution holds the potential to provide key information for identification of prognostic and diagnostic markers as well as the development of precise and personalized cancer treatment.

## Introduction

Colorectal cancer (CRC) is among major cancer worldwide in terms of incidence and mortality, with increasing trend, particularly in developing countries (Granados-Romero et al., 2017). According to global cancer statistics 2018, CRC was the third most commonly diagnosed cancer (10.2% of total cases) and ranked second for cancer-related deaths (9.2% of the total cancer deaths) (Bray et al., 2018). Approximately 30 to 50% of newly diagnosed patients will progress into metastatic CRC (mCRC) with 5-year survival rate of 50 to 60% (Arvelo, 2015; Engstrand et al., 2018). Despite the advancement in cancer detection tools and treatment options, metastasis remains a hindrance for effective treatment (Chakraborty and Rahman, 2012). Over the years, several mechanisms have been proposed to explain the metastatic progression in CRC, mainly *via* disturbance of cellular processes, epigenetic modifications, and genomic alterations (Herceg and Hainaut, 2007; Wong et al., 2007; Kanwal and Gupta, 2012; Arvelo, 2015). Aside from conventional treatment, in particular, chemotherapy (5-Fluorouracil, Oxaliplatin, Irinotecan and Capecitabine), many new targeted agents are also available for metastatic CRC (mCRC), including vascular endothelial growth factor (VEGF)-targeted therapy (Bevacizumab) and anti-epidermal growth factor receptor (EGFR)-targeted therapy (Cetuximab and Panitumumab) (Riihimäki et al., 2016; Burz et al., 2018). Nevertheless, metastasis remains a challenge in treating CRC, and among the main reasons are mostly attributable to intratumoral heterogeneity (ITH) and the presence of circulating tumor cells (CTCs) (Worthley and Leggett, 2010; Séronie-Vivien, 2014).

Intratumoral heterogeneity (ITH) refers to the differences in genetic and molecular characteristics between cancer cells within a single tumor or due to the various degrees of cellular differentiation (Punt et al., 2017), whereas precision treatment, often called personalized treatment, exploits patient’s as well as cancer-specific molecular and pathologic signatures to target cancerous cells (Xue and Wilcox, 2016). However, in an actual scenario, not only did these precision therapies remain unresponsive to a significant amount of patients, but also promote acquired drug resistance if inhibitors were added to maximize cancer cell death at initial stage, resulting in the rapid outgrowth of resistant clones and reoccurrence of CRC (Molinari et al., 2011). One plausible explanation to this matter was that current precision medicine was tailored based on transcriptome analyses, which utilized bulk tumor data but lacked the ability to capture ITH (Valdes-Mora et al., 2018). The presence of ITH, in turn, obscured precision cancer treatment (Hutchinson, 2014; Seoane and De Mattos-Arruda, 2014). Hence, studying the cancerous cells in single-cell resolution, at molecular level, in order to understand ITH, is necessary for precision therapy and the prediction of therapeutic efficacy (Punt et al., 2017). With the recent development of high-throughput single-cell RNA sequencing (scRNA-seq), scientists now have the power to dissect the diverse cellular populations of cancers (Bagnoli et al., 2019). In the future, it is possible that the scRNA-seq technique is applied to guide the selection of targeted combination therapies and assist in determining the enrolment criteria for clinical trials.

## Single Cell Transcriptome Analysis in CRC

At present, transcriptome analyses have been intensively applied to understand the heterogeneity of tumors *via* examining the gene expression level (mRNA) present in bulk tumor cell populations (Marisa et al., 2013; Sadanandam et al., 2013; Sadanandam et al., 2014). The two most recent advances in molecular pathological classification systems for CRC are The Cancer Genome Atlas (TCGA) (The Cancer Genome Atlas Network, 2012) and Consensus Molecular Subtypes (Guinney et al., 2015). The classification systems are capable of classifying tumors into subgroups with distinct molecular and establishing signatures/clinical features to predict treatment response and patient outcomes (Budinska et al., 2013; Roepman et al., 2014). Nonetheless, the overall progress is still largely hindered because of the limitation of these bulk profiling technologies in capturing ITH (Seoane and De Mattos-Arruda, 2014). Therefore, there has been rising attention in the study of single-cell transcriptomics which is capable of examining the expression levels of individual cells within a given population.

Single-cell sequencing is a powerful technology for investigating ITH by identifying genomic alterations and distinct transcriptomic states in single tumor cells (Patel et al., 2014). To date there are only a few published studies on single-cell transcriptomes of CRC. One of the first studies was published in 2017 by Li and his colleagues, which includes 11 primary CRCs (375 cells) and matched normal mucosa (215 cells) (Li et al., 2017). Single-cell RNA sequencing was performed on 969 resected primary tumor cells from 11 CRC patients, and 622 single cells from the nearby normal mucosa of seven of the patients. The authors developed a novel clustering method, named reference component analysis (RCA) and obtained seven distinct cell clusters, which were annotated as epithelial cells, fibroblasts, endothelial cells, B cells, T cells, mast cells and myeloid cells. Interestingly, although the differentially expressed genes identified by scRNA-seq and bulk analyses were significantly concordant, the majority of differentially expressed genes from scRNA-seq were undetected in bulk analysis. Epithelial–mesenchymal transition (EMT)-related genes were upregulated only in the cancer-associated fibroblast subpopulation of CRC samples. Their results indicated that *via* projecting bulk-tumor transcriptomes onto single-cell transcriptomes, existing CRC classification system could be further refined.

In another attempt to reveal CRC tumor heterogeneity, Ono and her coworkers combined single-cell DNA and RNA sequencing technologies with a mouse CRC model, ideal for time-series analysis (Ono et al., 2019). Single-cell exome and transcriptome sequencing of 200 cells were performed to identify ITH from one single cell. The authors demonstrated that mouse cancer cells, after undergoing alteration in transcriptional and genetic ITH, can adapt to the drastic environmental changes of allograft into a mouse. During this process, new subpopulations of cells, showing mesenchymal–epithelial transformation (MET), were generated. In addition, human CRC data from TCGA revealed a remarkable trend of metastasis in a fraction of human patients whose expression patterns were similar to those of the mouse-cell subpopulations. In a nutshell, their study revealed an evolutionary pattern of single-cell RNA and DNA changes in cancer progression and a superior CRC classification based on its ITH. **Table 1** summarizes the recent findings from single-cell transcriptome studies in CRC.

**Table 1:** Summary of recent findings of single-cell RNA-seq and bulk analysis of CTCs in CRC.

Transcriptome	Type	Sample	Finding	Citation
Single cell analysis	Primary CRC	Primary CRC cells from CRC patients	Development of reference component analysis (RCA) which obtained seven distinct cell clusters (epithelial cells, fibroblasts, endothelial cells, B cells, T cells, mast cells and myeloid cells).	(Li et al., 2017)
		Organoids from primary CRC human tumors (stage 1)	Detection of four newly emerged chemoresistant cell subtypes (c29, c30, c31, and c32) after Oxaliplatin treatment with different drug responses.	(Chen et al., 2018)
		2824 primary CRC cells from a CRC patient	Detection of five distinct cell clusters from a CRC patient with clear sign of heterogeneity, where each cluster consisted of specific cell markers with different functions.	(Dai et al., 2019)
	Metastatic CRC	Metastatic single cells from CRC patients (stage III or IV)	Confirmation of the feasibility of genetic lineages reconstruction together with their epigenomic and transcriptomic dynamics.	(Bian et al., 2018)
		CRC mouse model and metastatic mouse cancer cells	Validation of the dynamics of ITH in mouse CRC models and relate to CRC in humans *via* comparison with TCGA data.	(Ono et al., 2019)
		Samples of liver metastasis cancer tissue and adjacent tissue from CRC patients	Identification of a total of 12 clusters corresponding to 6 cell types, including cancer cells, T cells, myeloid cells, endothelial cells, fibroblasts and B cells from patient sample of CRC liver metastasis.	(Zhang et al., 2019)
	CRC cell line	Secondary cell line (HCT116 cells)	Identification of multiple adaptive resistance mechanisms to regorafenib in CRC *via* single cell RNA sequencing.	(Sathe et al., 2019)
Bulk analysis	Metastatic CRC	EpCAM-based immunoisolation of CTC from 6 mCRC patients	Identification of 410 genes related to cell movement and adhesion, cell death and proliferation, and cell signaling and interaction *via* cDNA microarray.	(Barbazán et al., 2012)
		CTC lines from the blood of 3 advanced mCRC patients	Validation of genetically and phenotypically heterogeneity in CTC lines. Identification of gene subset commonly enriched in cultured CTC and CTCs from colon and other cancers. CTC lines expressed high levels of drug metabolism genes and were resistant to conventional therapies.	(Grillet et al., 2017)

## The Necessity of Single-Cell Transcriptome Analysis in Metastatic CRC

Majority of CRC-related deaths were related to metastatic progression (Riihimäki et al., 2016). The high metastatic rate of CRC (approximately 30 to 50%) exacerbated the situation (Engstrand et al., 2018). In this context, there is an uprising interest in the discovery of new target agents for cytotoxic drugs. However, there are limited approved targeted therapy for treating mCRC, and some of these examples are EGFR monoclonal antibodies (mAbs), VEGF mAbs, anti-VEGF receptor-2 mAbs, recombinant fusion protein (Zivaflibercept) and oral multikinase inhibitor (Regorafenib). The former is available for RAS wild-type patients, while the remaining are effective for those with RAS mutation (Martini et al., 2017). Unfortunately, all these treatments are unresponsive towards CRC with alterations in genes such as *BRAF* and *PIK3CA*, posing a greater risk to patients with these subtypes (Sartore-Bianchi et al., 2009; De Roock et al., 2010; Tamborero et al., 2018). Thus, new targets for drug development and techniques in identifying the complex molecular heterogeneity of mCRC, in particular, are urgently needed (Lim et al., 2019).

Single-cell transcriptomics is most likely to contribute more specific diagnostic and prognostic markers, and actionable therapeutic targets for personalized cancer medicine than bulk transcriptomics (Zhang et al., 2014). However, it was limited in terms of characterization of multiple layers of molecular features in each genetic lineage. Therefore in the year 2018, a powerful scTrio-seq (single-cell triple omics sequencing) technique was established, which was capable of examining mutations, transcriptome, and methylome simultaneously from a single cell. CRC tumors and metastases from 10 individual patients (stage III or IV) were subjected to scTrio-seq, and their analysis provided insights into tumor evolution linked DNA methylation to genetic lineages and confirmed that DNA methylation levels were consistent within lineages but can differ substantially among clones (Bian et al., 2018). To summarize, all recently conducted research converged to the necessity to tailor individualized cancer treatment based on the analysis of gene expression patterns at single-cell resolution, which hold the potential to shed light on a key mechanism behind the development of metastasis.

## Possible Solution to CRC Chemoresistance

Existing advances in cancer treatment fall short of offering an adequate solution to chemoresistance, especially among patients at the advanced stages of CRC (Hammond et al., 2016). The formation of chemoresistant cells is frequently attributed to the presence of rare drug-resistant clones in the tumor before or after treatment (Shi et al., 2018). In general, these chemoresistant subtypes of CRC attain the ability to disrupt drug transport, dysregulate cellular processes, alter drug sensitivity (via genetic or epigenetic modifications) and targets of therapy, that subsequently limit the efficacy of existing anti-cancer therapies (Holohan et al., 2013; Panczyk, 2014; Hu et al., 2016; Zhang and Wang, 2017; Hon et al., 2018; Abu et al., 2019). Since there are hints that metastasis and chemoresistance can be interconnected (Zheng, 2017; Durinikova et al., 2018), the previous can be prevented if chemoresistant subtypes are identified early for optimal or more aggressive treatment. Unfortunately, the mechanisms responsible for chemotherapy resistance by CRC have not been clearly identified. Moreover, current chemotherapy does not possess the strength to fully eradicate solid tumors, resulting in secondary tumor and relapse. Owing to this, numerous efforts have been made to dissect the chemoresistant cancer cells based on the genes expressions, epigenetics, pathways signatures and therapeutic responses (Datta et al., 2016; Baharudin et al., 2017; Abu et al., 2019; Li et al., 2019).

Although bulk transcriptomics is adequate to study the average gene expression signatures related to chemoresistance, they generally involve bulk tissue with assumption that all the cells obtained are of homogeneous material, thereby ignoring the stochasticity of gene expression (Raj and van Oudenaarden, 2008; Stegle et al., 2015). Single-cell transcriptomics analyses, on the other hand, are capable of studying the transcriptomes of individual cells and are more preferable for cell-specific precision therapies. For instance, a recent research by Chen et al. (2018) confirmed the capability of scRNA-seq in characterizing four different types of cellular subtypes from organoids, including drug-induced group (c29, c30, c31, and c32), drug insensitive group (c2, c5, c6, c7, c13, c22, c21, c24, c27, and c28), drug-sensitive group (c1, c3, c4, c8, c9, c11, c15, c19, c25, and c26), and drug ultrasensitive group (c10, c12, c14, c16, c17, c18, c20, c23), after treatment of Oxaliplatin. The first group displayed chemoresistance properties and appeared only after treatment. Studying of these subtypes would enable further detailed categorization based on the differential responses, genes and pathways involved, leading towards better therapeutic selection for CRC patients who might or already displayed chemoresistance before or after chemotherapy. Hence, scRNA-seq is foreseen to be applied to guide the selection of anticancer therapies and even in the prevention of chemoresistance in the future.

## CTC Characteristics as a Snapshot of Tumor Heterogeneity

**Figure 1:**
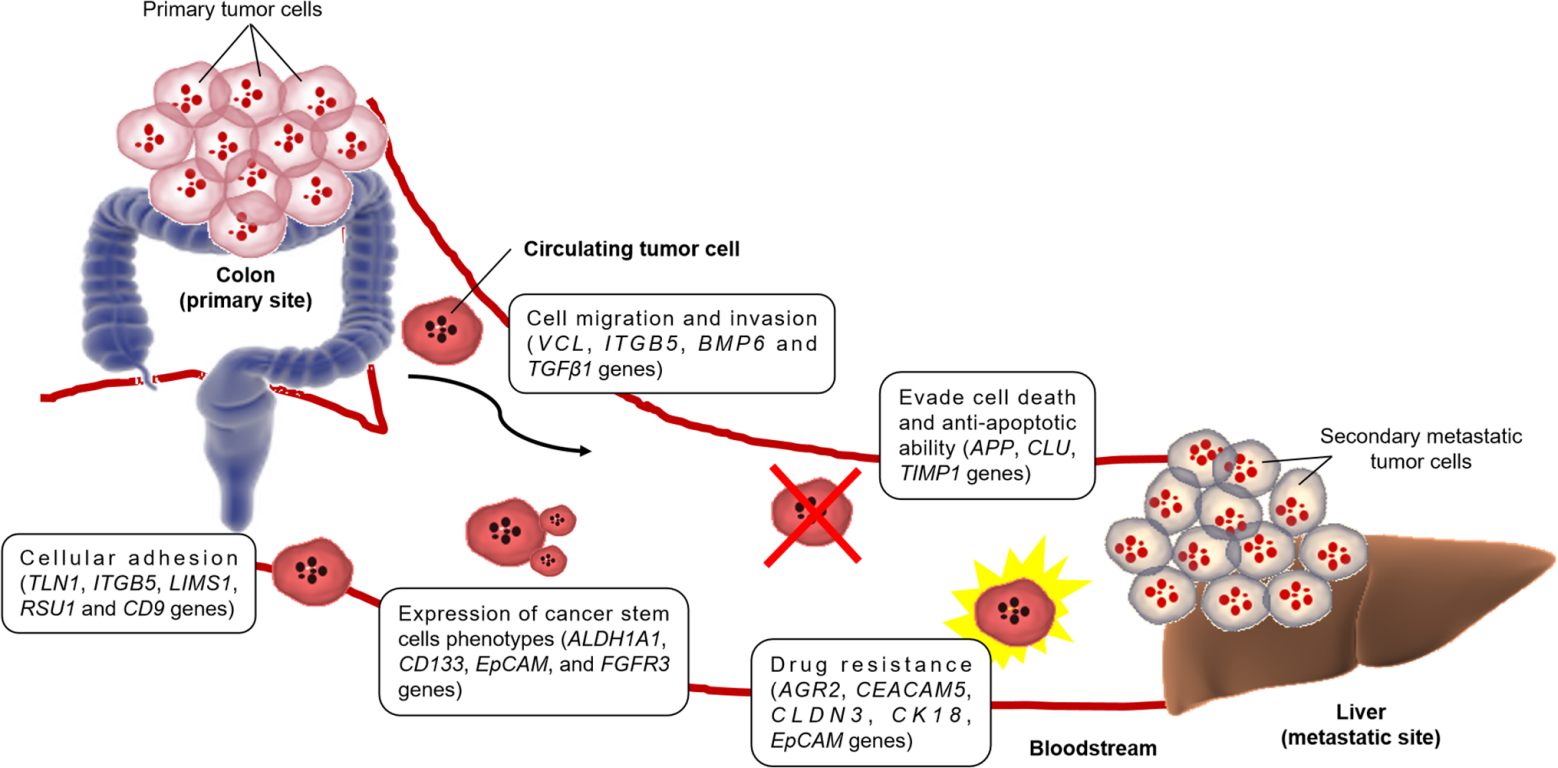
Figure representing mechanisms of distant metastatic colonization of CTCs and the genes involved in CRC (Barbazán et al., 2012; Grillet et al., 2017).

Circulating tumor cells (CTCs) are rare metastatic cells shed from the primary tumor into the circulatory system, forming secondary tumor at distant tissues (Ferreira et al., 2016) (**Figure 1**). According to the ‘seed and soil’ hypothesis by Stephen Paget in 1889, a seed (in this case, CTC) have the ability to form metastasis only in a location suited for this process (organs like liver, lungs), whereas the mechanistic theory based on the direction of blood flow from tumor proposed by James Ewing in 1920 assumes that potential of metastasis is dependent on drainage anatomy from the primary tumor. Both these two complementary hypotheses point out the potential of CTCs in causing metastasis during the progression of CRC. Since then, there have been several examples of validated clinical applications for CTC detection, proving its existence in CRC (Bork et al., 2015) and mCRC (Cohen et al., 2008), and also as a prognostic and predictive marker in CRC patients (Huang et al., 2014). As such, single-cell analyses of these CTCs are believed to provide critical insights into CRC cancer metastasis (Pantel and Alix-Panabières, 2012; Pantel and Speicher, 2016). However, characterization of CTCs from CRC, at the single-cell level, are as yet unknown. At present, there are only a few published papers on the bulk RNA sequencing analysis in CTCs.

As presumptive founders in the metastasis formation, CTC is becoming a field of interest, and the understanding of their biology may open new perspectives in oncology (Lim et al., 2019). In 2012, a group of Spain researchers performed molecular characterization of CTCs in human mCRC. Their objective was to investigate the biology of CTCs and improving their clinical utility in the CRC patients’ management (Barbazán et al., 2012). For this, EpCAM-based immunoisolation of CTC from six mCRC patients was combined with whole-transcriptome microarrays, revealing 410 genes related to cell movement and adhesion, cell death and proliferation, and cell signaling and interaction. All these genes characterized the CTC populations. Their study suggested CTCs as the diagnostic and prognostic biomarkers, which represented an innovative and promising approach in the clinical management of CRC patients in the foreseeable future.

Although CTCs have attracted a broad interest as potential markers of tumor progression and treatment response, the lack of functional characterization of these cells has become a bottleneck in taking these observations to the clinic. In addition, *in vitro* CTC models are lacking. Following this, Grillet and her coworkers generated several CTC lines from the blood of three advanced mCRC patients (Grillet et al., 2017). Characterization of these cells verified the presence of multipotent cells responsible for genetic and phenotypic heterogeneity, endowing them with strong metastatic potential. In addition, six genes (*AGR2*, *CEACAM5*, *CLDN3*, *KRT18*, *EpCAM* and *FGFR3*) were detected as differentially expressed in the generated CTC cell lines, which was similar to primary CRC cells grown under similar conditions (Smirnov et al., 2005; Mostert et al., 2015; Onstenk et al., 2015). The CTC lines also displayed enhanced drug/xenobiotics metabolizing activity, in particular *via* cytochrome P450 pathway, suggesting resistance to conventional therapies. To sum up, their study was the first experimental demonstration that CTCs isolated from mCRC patient could be used to determine drug sensitivity which aided in the formulation of personalized cancer medicine, even though the heterogeneity of CTCs was not investigated in single-cell resolution at molecular level.

## Challenges and Future Directions

Colon is a relatively large organ, categorized into four parts (the ascending colon, the transverse colon, the descending colon and the sigmoid colon) and is composed of multiple different cells (epithelial cells, stroma, muscle cells, fat, etc.). In order to obtain a single-cell suspension, the bulk tissue has to undergo mechanical or enzymatic dissociation. Isolating the single-cell suspension containing ‘healthy’ and contamination-free viable cells are indeed a challenge for scRNA-seq studies. Moreover, there are several technical limitations in scRNA-seq like the number of cells that can be studied at a time and the depth of sequencing required. The higher the number of cells, the higher the sequencing depth needed, hence increasing the overall cost of scRNA-seq experiment. If the number of cells must be limited to a certain amount, then the overall representative of the single-cell transcriptome will be questioned. Therefore, CTCs are believed to be the versatile components which warrant a spotlight in CRC research in order to identify biomarkers that will benefit the metastatic or chemoresistant CRC patients, while providing representative data. Also, scRNA-seq requires the cells to be intact after sorting or enrichment, prior to library preparation to prevent RNA degradation. The ability of CTCs to survive from harsh environments in the bloodstream makes them the ‘tough’ cells and the best candidate for scRNA-seq in studying CRC (Steinert et al., 2014).

At present, various technologies are developed for CTCs isolation, however, they are mainly used for research purposes rather than clinical applications (Bankó et al., 2019). Among the obstacles are the presence of ITH (Levitin et al., 2018; Lim et al., 2019), limited knowledge on CTCs mechanism of action in cancer progression (shedding from the primary/metastasis tumor, survival in bloodstream, avoidance of apoptosis, colonization potentials and settlement in distant organs), the rarity of CTCs (0 to 10 CTCs/ml whole blood in 30% to 50% mCRC patients) (Zieglschmid et al., 2005), various sizes of CTCs and the lack of clinical validation (Millner et al., 2013; Rejniak, 2016; Kowalik et al., 2017; Bankó et al., 2019). In addition, there are only a few studies relating to CTCs pharmacogenomics and underlying survival mechanisms (Wang et al., 2018) as well as the cell-cell interactions in CRC microenvironment (Krog and Henry, 2018), and majority of them are based on experimental and theoretical extrapolations (Burz et al., 2018). For instance, Yu et al. (2014) suggested that pharmacogenomic profiling of invasive CTCs could predict chemotherapy response and resistance, whereas Steinert et al. (2014) identified upregulation of *CD47* in concordance with the mark down-regulation of calreticulin, which were believed to mediate immune escape and survival mechanisms of CTCs in CRC. In 2016, Ning et al. (2018) proposed CTCs as a clinically useful prognostic marker in mCRC patients as they displayed Akt-2 expression that mediated epithelial mesenchymal transition. Although CTCs demonstrated potential as a predictive marker (Yap et al., 2014) and matched most of the cancer hallmarks described by Hanahan and Weinberg (Hanahan and Weinberg, 2011; Hanahan and Weinberg, 2017; Fouad and Aanei, 2017), current understanding about their pharmacological and clinical knowledge is still limited. Therefore, we anticipate that, in the near future, with the advancement of single cell technologies, CTCs could be proven useful in CRC management and a routine screening for cancer patients.

Another bottleneck in scRNAseq is the bioinformatics data analysis. Various algorithms have been developed to infer cell types by clustering scRNAseq profiles, however, a robust algorithm is yet to be developed due to high noise levels, technical variability and batch effects (Gao, 2018; Chen et al., 2019; Choi and Kim, 2019). To date, gene expression in such tumors has been profiled using bulk transcriptome methods, providing a single transcriptome measure that represents many cell types (Barbazán et al., 2012; Grillet et al., 2017). By employing single-cell transcriptomic technology, it is now possible to deconstruct a tumor into its component cell-type parts and therefore gain a better understanding of the underlying biology. In conclusion, the analysis of gene expression patterns of CTCs from CRC at single-cell resolution holds the potential to provide key information for identification of prognostic and diagnostic markers as well as the development of precise and personalized cancer treatment.

## Author Contributions

N-SM is the principal investigator that conceptualize the idea of this manuscript. FT, RB and N-SM wrote the manuscript, while R-IM, NA and L-HL provide critical insights.

## Funding

This review is supported by Dana Impak Perdana Grant (DIP-2018-010) by Universiti Kebangsaan Malaysia (UKM).

## Conflict of Interest

The authors declare that the research was conducted in the absence of any commercial or financial relationships that could be construed as a potential conflict of interest.

## Abbreviations

CRC, Colorectal cancer; CTC, Circulating tumor cell; DNA, Deoxyribonucleic acid; ITH, Intratumoral heterogeneity; mCRC, Metastatic colorectal cancer; RNA, Ribonucleic acid; scDNA-seq, Single cell DNA sequencing; scRNA-seq, Single cell RNA sequencing; scTrio-seq, Single-cell triple omics sequencing.
